# Immunotherapy-Associated Cardiotoxicity: Current Insights and Future Directions for Precision Cardio-Oncology

**DOI:** 10.3390/cancers17172838

**Published:** 2025-08-29

**Authors:** Eleni Stefanou, Georgios Tsitsinakis, Dimitra Karageorgou, Christo Kole

**Affiliations:** 1 Artificial Kidney Unit, General Hospital of Messinia, 24100 Kalamata, Greece; 2 Cardiology Department, Sismanogleio General Hospital of Attica, 15126 Athens, Greece; 3 Faculty of Medicine, National and Kapodistrian University of Athens, 15784 Athens, Greece

**Keywords:** immunotherapy, cardiotoxicity, immune-related adverse events, myocarditis, arrhythmia, heart failure, artificial intelligence, genetic profiling

## Abstract

**Background/Objectives**: Cancer immunotherapy has revolutionized the field of oncology by harnessing the immune system to attack cancer cells, increasing survival in a broad spectrum of malignancies. However, despite its positive therapeutic benefit, immunotherapy is also associated with a spectrum of adverse events affecting various vital organs, including the cardiovascular system. **Methods**: We conducted a comprehensive review of the available literature on the epidemiology, pathophysiological mechanisms, and current management approaches for cardiovascular adverse events associated with cancer immunotherapy. In addition, we evaluated emerging personalized strategies and interventions aimed at mitigating these risks and improving patient outcomes. **Results**: Immunotherapy is associated with a broad spectrum of potentially serious cardiovascular adverse events, including immune-mediated myocarditis, heart failure, arrhythmias, pericarditis, and accelerated atherosclerosis. Among these, immune checkpoint inhibitor-associated myocarditis is the most well characterized and potentially fatal form of cardiotoxicity, with reported mortality rates approaching 50%. Similarly, chimeric antigen receptor T-cell therapy, despite its powerful antitumor efficacy, is frequently associated with cytokine release syndrome—a profound immune activation that can lead to significant systemic and cardiovascular complications. In response to these challenges, several personalized strategies are currently under development, including artificial intelligence and machine learning approaches, genetic and transcriptomic profiling, novel biomarker discovery, and integrated risk scoring systems, all aimed at enhancing risk stratification and improving patient care. **Conclusions**: Cancer immunotherapy has been associated with a range of immune-related cardiac adverse events, both non-severe and severe. As such, it is critically important to adopt a personalized approach to patient management before, during, and after the administration of immunotherapy. Early recognition through heightened clinical vigilance, along with the implementation of individualized risk assessment tools, is essential for identifying patients at high risk of immunotherapy-induced cardiotoxicity. These strategies are imperative for optimizing patient outcomes and ensuring safe and effective cancer treatment.

## Simple Summary

Cancer immunotherapy has revolutionized the field of oncology by harnessing the immune system to attack cancer cells, increasing survival in a broad spectrum of malignancies. However, despite its positive therapeutic benefit, immunotherapy is also associated with a spectrum of adverse events affecting various vital organs, including the cardiovascular system. Cardiotoxicity secondary to immunotherapy includes myocarditis, atherosclerosis, pericarditis, arrhythmias, and cardiomyopathy, which may be life-threatening to some patients. Therefore, high suspicion for early recognition as well as the use of personalized tools for the identification of patients who are vulnerable to developing cardiotoxicity is imperative to prevent these adverse events. Our work summarizes the current knowledge on the epidemiology, pathophysiological mechanisms, and purposed management of cardiovascular toxicities linked to immunotherapy. In addition, it also highlights new personalized strategies—including biomarkers, artificial intelligence, and genetic profiling—aimed at predicting and preventing these complications to improve outcomes for cancer patients.

## 1. Introduction

In recent years, the use of novel therapies and personalized care, such as immunotherapy, in the treatment algorithm of many tumors has marked a therapeutic renaissance in oncology [1,2]. Immunotherapy is a therapeutic process that increases or restores the ability of the immune system to detect and destroy cancer cells by modifying and/or blocking co-stimulatory signals [3,4] or other steps in the cancer–immune cycle. Types of immunotherapy include immune checkpoint inhibitors (ICIs), which consist of monoclonal antibodies that selectively bind to and inhibit immune checkpoints; immunosuppressive molecules located on the membranes of antigen-presenting cells (APCs); and CD4^+^ T lymphocytes. These molecules play a key role in modulating immune activation levels and preventing the onset of autoimmune reactions [5], therefore blocking these molecules from activating T cells and promoting elimination of cancer cells [6]. Other types of immunotherapies include therapeutic cancer vaccines, a form of active immunotherapy that delivers cancer target antigens, including tumor peptides, whole-cell tumor vaccines, DNA agents, or oncolytic viruses; dendritic cell vaccines to trigger or amplify the host immune system to recognize and eliminate cancer cells [7,8,9]; and Adoptive Cell Transfer, involving autologous or allogeneic graft of tumor-infiltrating lymphocytes or ex vivo genetically modified T cells expressing novel T-cell receptors or chimeric antigen receptors [10,11]. Currently, three types of ICIs have been approved by the FDA for cancer treatment, such as programmed death-1 (PD-1) inhibitors, including pembrolizumab, nivolumab, cemiplimab; its ligand (PD-1L), including atezolizumab, avelumab, and durvalumab; ipilimumab and tremelimumab, which are cytotoxic T-lymphocyte antigen 4 (CTLA-4) inhibitors; and relatlimab, a lymphocyte activation gene-3 (LAG3) inhibitor [12,13]. CAR T-cell therapy (CAR-T), which involves the patient’s own ex vivo-modified T cells to target specific antigens [14]; and bispecific T-cell engager (BTE) therapy, consisting of non-IgG-like bispecific antibodies designed to bind to both CD3 and tumor-associated antigens [15], are also FDA-approved therapies for specific patient populations [16].

Despite its positive therapeutic benefit, immunotherapy is also associated with a spectrum of immune-related adverse events (irAEs) affecting various vital organs, including the cardiovascular (CV) system [17]. In this review, we use the term cardiovascular adverse events (CV-AEs) to refer to clinically significant complications involving the heart and vasculature. Cardiotoxicity secondary to immunotherapy briefly includes complications such as myocarditis, atherosclerosis, pericarditis, arrhythmias, and cardiomyopathy, which often may be so serious and even life-threatening that they can result in the discontinuation of the immunotherapeutic treatment [18,19]. Consequently, the ability to elucidate and individualize cardiovascular risk stratification prior to initiation of therapy, the identification of patients who may be vulnerable to developing such CV-AEs, and increased suspicion for early recognition and management of these patients when CV-AEs occur are of high importance. In the present work, we critically evaluate the current literature on the epidemiology, pathophysiological mechanisms, and the purposed management of cardiotoxicities associated with the different types of immunotherapy. In addition, we discuss emerging personalized strategies aimed at predicting and reducing cardiovascular complication events in patients receiving immunotherapy, with the goal of increasing the benefit of cancer therapy.

## 2. Methods

This work was conducted as a comprehensive narrative review of the current literature to synthesize current knowledge on immunotherapy-associated cardiotoxicity. A structured search strategy was applied across PubMed, Scopus, Web of Science and ClinicalTrials.gov databases from January 2010 through May 2025. Search queries included combined controlled vocabulary (e.g., MeSH terms) and free-text keywords, including the following: “immunotherapy”, “immune checkpoint inhibitors”, “Adoptive Cell Transfer”, “therapeutic cancer vaccines”, “Anti PD-1L”, “anti-PD-1”, “anti-CTLA-4”, “CAR-T”, “bispecific T-cell engagers”, “cardiotoxicity”, “cardiovascular adverse events”, “myocarditis”, “arrhythmia”, “pericarditis”, “heart failure”, “Cardiomyopathy”, “Vasculitis”, “Myocardial Infraction”, “Ischemic Stroke”, “Cardiogenic shock”, and “Cardiac Death”.

Eligible sources included clinical trials, observational studies, registry analyses, pharmacovigilance reports, case series, and mechanistic studies that reported cardiovascular outcomes in the context of immunotherapy with a focus on publications in English. Only peer-reviewed articles in English were included. Studies that exclusively addressed non-cardiovascular toxicities or preclinical animal models without translational relevance were excluded. Reference lists of eligible articles and recent reviews were screened to identify additional relevant publications.

The goal of this work was to provide an integrated overview rather than a formal systematic review; therefore, PRISMA methodology and risk-of-bias tools were not applied. Instead, evidence was critically appraised and synthesized according to study design, population, and clinical relevance, with the aim of highlighting epidemiological patterns, mechanistic insights, and evolving strategies for precision cardio-oncology.

## 3. Immune Checkpoint Inhibitor-Associated Cardiovascular Adverse Events

Immune checkpoint inhibitors (ICIs) are generated antibodies that bind selectively and block the checkpoint proteins PD-1, PD-L1, and CTLA-4 expressed by tumor cells to escape the T cell-mediated antitumor response, therefore allowing T cells to recognize and eliminate cancer cells [20]. Both PD-1 and PD-L1 are also expressed normally in other tissues, including human cardiomyocytes. Inhibition of these checkpoint proteins can lead to infiltration of CD4+ and CD8+ T cells, as well as macrophages, in the myocardium, which has been associated with lymphocyte-mediated cardiac toxicity [21]. This mechanism is also highlighted by results in preclinical models [22,23,24,25]. ICI-related CV-AEs (Table 1) are rare, occurring in approximately 1 to 1.5% of the patients treated with immune checkpoint inhibitors; however, they are potentially fatal, with an estimated mortality rate of up to 50% in the case of myocarditis [26,27,28] and lower mortality rates, 21% and 6%, in the case of pericarditis or vasculitis, respectively [29,30]. Therefore, they require a high index of suspicion by treating clinicians. Other ICI-related CV-AEs include acute coronary syndromes and thromboembolic events; arrhythmias; and left ventricular (LV) dysfunction, including takotsubo syndrome (TTS), also known as stress-induced cardiomyopathy, without any evidence of myocarditis have also been reported [18,26,31,32,33,34,35,36,37].

**Myocarditis** refers to an inflammatory disease of the myocardium caused by a variety of infectious (viral, bacterial, protozoan parasites) and noninfectious factors, including hypersensitivity reactions, systemic disorders, toxins, and radiation [38]. Myocarditis is the most commonly considered cardiovascular irAE with ICI therapy, with the incidence ranging from 0.5 to 1.7% [39] occurring at a median time of 34 days (21 to 75 days) after initiation of ICI treatment, with a wide variation of 2 to 454 days [25,28,31]. Cases of later myocarditis, nevertheless, have also been described up to several months after initiation of therapy [40]. The incidence of myocarditis in patients receiving single-agent ICI therapy, nivolumab, was reported at approximately 0.06% [41] and 0.27% to 1% for patients receiving a combination of ICIs (Table 1 and Table S1) [26,28,39,41,42,43,44,45]. Other analyses, such as the World Health Organization’s (WHO) Global Individual-Case-Safety-Report database [25] and a prospective observational surveillance study, reported a rate of 1.4% of myocarditis for single-agent treatment [46] and 1.7% to 2.4% for a combination ICI treatment, such as relatlimab (anti-LAG-3) plus nivolumab (anti-PD1) and ipilimumab (anti-CTLA4) plus nivolumab [28,39]. It is reported that ipilimumab plus nivolumab increases the risk of myocarditis by 4.74-fold compared to standalone therapy [41]. These results were also supported by a meta-analysis of 22 clinical studies [47]. Differences have also been observed among different ICI regimens. Interestingly, a pharmacovigilance study using the WHO’s global database found an incidence of 0.41% of all case safety reports for anti-PD-1/PD-L1 compared to 0.07% for anti-CTLA-4 agents, with an odds ratio (ROR) of 5.62 (95% CI: 2.46–12.88) [26]. These results were further supported by a meta-analysis reporting an incidence of 0.57% for anti-PD-1 compared to 0.16% for anti-CTLA-4 [19]. Patients usually present with impaired left ventricular ejection fraction (LVEF) with an incidence of 12.3 ± 2.7%, while a reduction in global longitudinal strain (GLS), a sensitive marker of myocardial dysfunction [48], was observed in 14.1 ± 2.8% (*p* < 0.001) of patients under ICI therapy [44]; other common complications of myocarditis, such as conduction abnormalities, arrhythmias, and heart failure, are also reported [49]. As reported above, the prognosis in patients with ICI-induced myocarditis is significantly worse, with mortality rates ranging from 25 to 50% compared to 4% in non-ICI-induced myocarditis during a median follow-up of 4.7 years [28,39,50].

**Table 1:** Immune checkpoint inhibitor-associated cardiovascular adverse events.

Adverse Event	Incidence Among All Reported irAEs	Incidence Among Cardiovascular Toxicity	Key Findings—Comments	Studies
Myocarditis	0.5–1.7%; up to 2.4% with combination therapy	79–100%	Mortality: 25–50%; Median onset: 34 days; Treatment: Immunosuppressants; high vigilance required, according to guidelines	[28,31,39,41]
Cardiomyopathy/ Heart failure		Takotsubo cardiomyopathy: 14%; heart failure: 1.6%	Treatment: According to guidelines	[31,51]
Pericarditis/Pericardial Disease	0.36% to 1.57%	21%	Mortality: 21% Treatment: Pericardiocentesis; supportive care; according to guidelines	[26,32,52]
Vasculitis	0.26%	19%	6% mortality in some studies; none in others Treatment: According to guidelines	[26,53]
Atherosclerotic Events		Myocardial infarction: 0.95–7%; ischemic stroke: 0.91–7%	Mortality: Not specified; Treatment: Statins/corticosteroids (attenuated plaque progression)	[18,54,55,56]
Arrhythmias	<1%	Atrial fibrillation: 30%; supraventricular arrhythmias: 50%; conduction disorders: 17%; ventricular arrhythmias: 27%	Mortality: Not specified Treatment: Monitoring; likely anti-arrhythmic drugs; arrhythmia treatment according to guidelines	[26,31,57]
Valvulitis	Rare; case reports		Mortality: Not specified	[58]
Hypertension	13.2% compared to the non-ICI group (9.7%)		Recent meta-analysis did not find any significant increase	[59,60]

irAEs: immune-related adverse events; ICI: Immune checkpoint inhibitors.

**Pericardial disease** refers to an inflammatory disease of the pericardium, a double-layered, fibroelastic sac surrounding the heart and separated by a space that normally holds 15 to 50 mL of serous fluid [61,62]. ICI-induced pericardial disease can also be associated with myocarditis. Clinical manifestations are represented by pericarditis accompanied by no or mild pericardial effusion, or even cardiac tamponade [63,64]. The precise mechanism underlying immune checkpoint inhibitor (ICI)-associated pericardial disease remains unclear, as no dedicated studies have definitively elucidated it. However, it is hypothesized that the condition arises from T-cell activation induced by ICIs, resulting in immune-mediated inflammation of the pericardium [26]. The incidence of ICI-mediated pericardial disease, including pericarditis and pericardial effusion, is reported to be from 0.36% to 1.57% [26,32] and associated with a 1.5-fold mortality risk [52]. Hemodynamically significant pericardial effusions were reported in 0.38% of patients [65]. Pericarditis and myocarditis can occur concomitantly or on their own [66]. In 88,928 identified ICI patients, the incidence of pericarditis (0.22%) and cardiac tamponade (0.47%) was less than 1%, while pericardial disease occurred in 4.99%, with pericardial effusion in 4.71% of patients (Table 1 and Table S1) [66]. As with myocarditis, pericarditis due to anti-PD-1 or anti-PD-L1 monotherapy occurred more frequently compared to anti-CTLA-4 (0.36% and 0.16%, respectively), with an ROR of 2.28 (95% CI: 1.27–4.12) [26]. Pericarditis is reported to develop 6 weeks and 11 months after starting ICI treatment, with one exception, and one in five cases occurs as early as 4 days after starting ICI treatment, with pericardiocentesis being the main treatment option for this patient. Analysis of the pericardial fluid showed the presence of white blood cells, mostly lymphocytes, and no signs of cancer [64,67,68,69,70].

**Vascular toxicity**, including vasculitis, atherosclerosis-related events, and arterial/venous thrombosis, accounts for 0.26% of all reported irAEs, but the exact incidence remains unknown [18,26,33]. The exact mechanism remains unknown, although emerging data propose that ICIs might accelerate atherosclerosis and promote atherosclerotic cardiovascular toxicity through changes in plaque composition [71,72,73,74]. Drobni et al. reported that the rate of progression of total aortic plaque volume in patients receiving ICI therapy was more than three-fold higher compared to the control group and was partially smaller when there was concurrent use of statins or corticosteroids [18]. Salem et al., in an observational, retrospective, pharmacovigilance study, reported a mortality rate of 6% in patients with ICI-associated vasculitis [26], while in another study, no related deaths were observed [53]. Acute vascular events within six months after ICI initiation were also reported in 2.6%, with (95% CI 1.8–3.6) [54]. These results were confirmed by a systematic review of 17 studies, consisting of a total of 10,106 patients following the administration of ICI therapy, which revealed a 1.1% incidence rate of arterial thrombotic events among ICI-treated patients [55]. Together, these data suggest that ACS may be another life-threatening cardiac adverse event occurring with ICI treatment [18,35].

**Arrhythmias** are another important complication that has been thought to result from underlying myocardial inflammation and is a harbinger of yet-to-be-diagnosed myocarditis [41]. Arrhythmias are observed to develop within 1 month of starting ICI treatment [75,76]. These arrhythmic complications, including atrial and ventricular tachyarrhythmias, as well as bradyarrhythmias, without any obvious myocarditis, are commonly observed [31,77,78,79]. It is proposed that the PD-1/PD-L1 pathway may have a direct participation in atrial fibrillation pathogenesis. PD-1/PD-L1 were found to be downregulated in atrial fibrillation patients compared to healthy controls, while patients with persistent atrial fibrillation were found to have lower levels of PD-1 than patients with paroxysmal atrial fibrillation [57]. Escudier et al. reported an incidence of atrial fibrillation, conduction disorder, and ventricular arrhythmia in 30%, 17%, and 27% of patients treated with ICIs, respectively (Table 1 and Table S1) [31], while in the WHO VigiBase Disproportionality analysis, supraventricular arrhythmias were reported in 50% of the patients [26]. In addition, in the Danish registry, arrhythmia was reported in 3.6 to 4.8% [51]. ICI-associated arrhythmias portend significant mortality, particularly sudden cardiac death [51]. At least one case of fatal ventricular arrhythmia was observed in the combination arm of an earlier phase II study of ipilimumab and nivolumab compared to ipilimumab alone [80].

**Other, less frequent ICI-associated adverse events**. Finally, a recent article in the European Heart Journal—Cardiovascular Imaging, in a case report of two patients treated with nivolumab and the second patient with nivolumab and subsequent addition of ipilimumab, both resulted in immunotherapy-mediated valvulitis. The first patient showed a progression of mitral and tricuspid regurgitation from mild to severe, while the second patient with trivial aortic regurgitation demonstrated a moderate-to-severe aortic regurgitation with evidence of valve thickening, as denoted by white arrow heads [58]. New onset of hypertension is also a reported side effect of ICI use, with the incidence being significantly higher in the ICI group (13.2%) compared to the non-ICI group (9.7%) [59]. However, a recent meta-analysis did not find any significant increase in the short-term risk of hypertension among patients treated with ICIs [60].

## 4. Chimeric Antigen Receptor T-Cell Therapy-Associated Cardiovascular Adverse Events

Chimeric antigen receptor T-cell (CAR-T) therapy is a type of Adoptive Cell Transfer therapy. While the pathophysiology of CAR T cell-associated cardiac dysfunction remains unclear, several potential mechanisms, including interleukin-6-mediated myocardial depression, cytokine release syndrome (CRS), and/or direct cardiotoxicity to off-target cross-reactivity of T cells, have been proposed so far [81,82]. CAR T-cells elicit a robust immune response characterized by a substantial systemic release of pro-inflammatory cytokines, including TNF-α, IFN-γ, and IL-6, which in turn activate prostaglandin pathways and precipitate CRS [83]. Preclinical studies have identified IL-6 and IL-1 as key mediators in the pathogenesis of CRS [84,85]. Interleukin-6 (IL-6) has been linked to a range of cardiovascular complications, including myocardial ischemia, atherosclerosis, heart failure, and hypertension [86]. The use of tocilizumab, an antibody targeting IL-6, reduces the effects of CRS and may have implications for reducing cardiotoxicity as well [87,88]. Characterized by fever and hypotension, CRS has traditionally been considered the key driver of cardiovascular toxicity associated with CAR-T and BTE therapies [89,90]. The American Society for Transplantation and Cellular Therapy (ASTCT) has established a consensus-based grading system for CRS [91]. Other proposed hypotheses include direct myocardial damage from recognition of cardiac antigens [92]. In a study by Linette et al., two patients died unexpectedly due to cardiogenic shock after receiving engineered autologous MAGE-A3-specific, HLA-A*01-restricted T-cell receptor gene engineered lymphocytes [82]. The autopsy revealed increased myocardial T-cell infiltration and myocardial damage. Despite the fact that MAGE-A3 is not expressed in myocardial cells, T-cell infiltration was attributed to off-target cross-reactivity against titin, a giant cytoskeletal protein found in striated cardiac muscle with a protein similar to the antigen on MAGE-A3 [82]. Although there are no large-scale studies on the multiple CV complications among adults treated with CAR-T therapies, small, cohort studies and case reports have shown that CV complications represent around 10–20% of total adverse events [87,93]. The most common CVEs reported to the FDA Adverse Events Reporting System were arrhythmia (77.6%), followed by HF (14.3%) and MI (0.5%), with a mortality rate of 30.1% for those reporting CVEs [94], while prolonged QTc has also been reported [95].

In addition, high-grade (3 and 4) cytokine release syndrome was strongly associated with increased risk of major adverse cardiac events occurring at a median of 11 days after the initiation of CAR-T cell therapy with a wide interquartile range (IQR = 6–151), underscoring a need for longitudinal cardiovascular monitoring [96]. Retrospective studies reported an incidence of heart failure (HF) (11–15%) and arrhythmia (1.4–12%) in patients treated with CAR-T cell therapy [87,97]. In addition, a comprehensive pharmacovigilance study, including 2657 patients treated with CAR-T therapy, reported tachyarrhythmias, 2.8%, adj.ROR = 2.78 (95% CI: 2.21–3.51); cardiomyopathy, 2.6%, adj.ROR = 3.51 (95% CI: 2.42–5.09); and pericardial diseases, 0.4%, adj.ROR = 2.26 (95% CI: 1.25–4.09), all IC_025_ > 0 [98], with atrial fibrillation found to be the leading tachyarrhythmia, followed by ventricular arrhythmias. A meta-analysis of thirteen studies, including a total of 1528 patients receiving CAR-T therapy with a median (IQR) duration of follow-up of 487 days, confirmed the high incidence of tachyarrhythmias and cardiomyopathy with a reported pooled prevalence for ventricular arrhythmia and supraventricular arrhythmia at 0.66% (95% CI: 0.00–2.28%) and 7.79% (95% CI: 4.87–11.27%), respectively [99]. In addition, left ventricular systolic dysfunction was reported at 3.87% (95% CI: 1.77–6.62%), heart failure events at 0.62% (95% CI: 0.02–1.74%), myocardial infarction 0.62% (95% CI: 0.02–1.74%; *I*^2^ = 57%), and 0.63% (95% CI: 0.13–1.38%) for cardiovascular death [99]. The pooled prevalence of all-cause mortality was 30.01% (95% CI: 19.49–41.68%) [99]. In addition, children and young adult populations receiving CD19-specific CAR-T cell therapy are also susceptible to cardiotoxicity, with a reported prevalence of 2.7–11% of left ventricular systolic dysfunction [100,101], and 17% developed hypotension requiring vasoactive agents [101]. Notably, 31% of patients with cytokine release syndrome had elevated troponin and reduced LVEF [101]. LV dysfunction, myocarditis, and arrhythmias are also associated with CD19-specific CAR-T therapy (Table 2 and Table S1). Concerning bispecific T-cell engager (BTE) therapy, to date, arrhythmias were the most frequently reported CV-AEs in major BTE trials, with the incidence ranging from 1.67 to 14%, followed by left ventricular systolic dysfunction [102,103,104].

## 5. Immunotherapeutic Vaccines

Adverse events, although low, still arise from the inflammatory response induced by Talimogene laherparepvec (T-VEC) or with other oncolytic viruses, causing mostly injection site redness or swelling, or on some occasions, flu-like symptoms, including fever, chills, and myalgia [111,112]. Studies on the cardiotoxic effects of anticancer vaccines are limited. In a study including 88 patients on T-VEC therapy, only one patient was reported to develop a grade 4 cardiac AE [113]. Another immunotherapeutic vaccine, sipuleucel-T, was associated with an increased risk of hypertension [114]. Sipuleucel-T is a personalized dendritic cell vaccine using an autologous product prepared from a patient’s own antigen-presenting cells that have been cultured with prostate antigen protein (PAP), which are prone to enhance the immune response to prostatic acid phosphatase antigen [115]. In addition, a case report of sipuleucel-T-induced inflammatory cardiomyopathy was also described after a review of the international pharmacovigilance database, VigiBase [114]. Moey et al. reported that patients with established cardiovascular risk factors who received sipuleucel-T are at a higher risk for developing congestive heart failure, myocardial ischemia, and/or supraventricular tachycardia. Therefore, electrocardiograms, as well as echocardiogram monitoring of the left ventricular function during weekly infusions, should be considered in these patients [114].

## 6. Risk Factors and Clinical Predictors of Immunotherapy-Associated Cardiotoxicities

Several key factors have been associated with increased risk of developing cardiotoxicity, including smoking; increased age (>75–80 years); arterial hypertension; diabetes; obesity; renal dysfunction; history of cardiovascular diseases, including coronary artery disease; heart failure; previous treatments with other chemotherapy regimens, such as anthracyclines or mediastinal radiotherapy; and the utilization of combined ICI therapy, and have been related with new development of heart disease or aggravate the already existing one (Table 3) [25,56,97,116,117,118,119]. A study of patients’ medical records receiving durvalumab, ipilimumab, nivolumab, and pembrolizumab at Wake Forest Baptist Health revealed that factors such as female gender, African American race, and smoking were significantly associated with an increased incidence of immunotherapy-associated cardiotoxicities [120]. In addition, a history of cardiovascular disease, such as acute coronary syndrome [56,121], aortic aneurysm [121], and hypertension [30,56,116,121], was also associated with increased incidence of ICI-mediated cardiovascular irAEs. As reported by Oren et al., the hazard ratios for ICI-induced myocarditis for history of acute coronary syndrome and for history of heart failure were 4.06 (95% CI, 1.15–14.3, *p* = 0.03) and 5.2 (95% CI, 1.4–18.7, *p* = 0.01), respectively, while for age it was reported at 1.07 (per each 1-year increase, 95% CI, 1.01–1.14, *p* = 0.02) (Table 3) [56]. These results were also supported by a meta-analysis, showing that 40% of all the reported cases that experienced cardiovascular irAEs had a previous history of cardiovascular disease, with myocardial infarction–peripheral coronary artery disease and hypertension being the most frequent reported key factors [19]. A history of cardiovascular disease and the presence of cardiovascular risk factors may predispose patients to developing CAR T-cell therapy-associated cardiomyopathy. Notably, most patients with a previous history of cardiomyopathy experienced grade ≥ 2 CRS and consequently required more intensive interventions, including tocilizumab administration (100% versus 67%, *p* = 0.017), vasopressor support (42% versus 8%, *p* = 0.004), and mechanical ventilation (25% versus 3%, *p* = 0.014), compared to patients without cardiomyopathy [97].

The relationship between diabetes mellitus and ICI-mediated myocarditis remains controversial. While two independent studies reported no significant association between diabetes mellitus and the development of myocarditis following immune checkpoint inhibitor (ICI) therapy [120,122], other studies have found a significantly higher prevalence of diabetes mellitus among patients with ICI-induced myocarditis compared to controls [56,123,124]. Moreover, data from an international registry identified combination immunotherapy, diabetes mellitus, obesity, and anti-CTLA-4 therapy as independent risk factors for ICI-associated myocarditis [28].

Additional factors associated with an elevated baseline risk of ICI-related cardiovascular toxicity include therapeutic regimens involving dual immune checkpoint inhibitor agents (e.g., ipilimumab combined with nivolumab), therapeutic regimens involving a combination of ICIs with other cardiotoxic agents, and a prior history of ICI-related adverse events [125,126]. A combination of ICIs with a chemotherapy regimen, especially with anthracyclines, which are highly cardiotoxic [127], significantly increases the incidence of cardiovascular irAEs, with a pooled risk ratio of 1.68 (95% CI: 1.07–2.64, *p* = 0.026), as shown by a systematic review and meta-analysis [19,128]. Supportive results were reported by another meta-analysis study [19]. Nevertheless, contradictory results were reported by a retrospective study by Bishnoi et al., showing that a combination of immune checkpoint inhibitors and chemotherapy is safe and does not result in increased cardiovascular toxicity, but instead showed lower hazards [129].

Additionally, in a study of a combination of ICI with vascular endothelial growth factor receptor (VEGF) inhibitor axitinib plus anti-PD-L1 avelumab, despite the increase in median progression-free survival in patients with PD-L1-positive tumors, the study was discontinued due to death attributable to myocarditis in the combination group that was included [130]. In addition, a meta-analysis reported a significant association with development of myocarditis in patients treated with both axitinib and pembrolizumab, ROR 36.9 (95% CI: 11.8–115.9), as well as with avelumab, ROR 55.6 (95% CI: 13.4–222.3) (Table 3) [131]. Taken together, these findings underscore the critical importance of comprehensive pre-treatment evaluation for pre-existing cardiovascular disease, comorbid conditions, and the specific type of cancer therapy—whether administered as monotherapy, in combination with other ICIs, or alongside other oncologic treatments—to ensure appropriate monitoring and management.

**Table 3:** Risk factors and clinical predictors of immunotherapy-associated cardiotoxicity.

Risk Factor	Study	Increased Risk	Studies
Age > 75–80	Increased susceptibility	ICI- and CAR-T-related cardiotoxicity 1.07 (per each 1-year increase, 95% CI, 1.01–1.14, *p* = 0.02)	[56,97]
Female sex	Wake Forest registry	ICI cardiotoxicity IRR 3.34 (95% CI 1.421, 7.849; *p* = 0.006)	[120]
African American race	Registry data	ICI cardiotoxicity IRR 3.39 (95% CI 1.141, 10.055; *p* = 0.028)	[120]
Smoking	Registry data	ICI cardiotoxicity IRR 4.21 (95% CI 1.289, 13.763; *p* = 0.017)	[116,120]
Hypertension	Strongly recurrent predictor	ICI myocarditis, CV irAEs	[30,56,116,121]
Obesity	Registry data/meta-analysis	ICI-induced myocarditis	[28]
Pre-existing CVD (CAD, HF, ACS, aortic aneurysm)	Registry data/meta-analysis	ICI-induced myocarditis 5.2 (95% CI, 1.4–18.7, *p* = 0.01) for history of heart failure, 4.06 (95% CI, 1.15–14.3, *p* = 0.03) for history of ACS CAR T-cell therapy-associated cardiomyopathy	[19,56,97,121]
Combined ICI therapy (PD-1 + CTLA-4)	Independent risk factor	ICI-induced CV irAEs	[25,125,126]
ICI therapy + chemotherapy (esp. anthracyclines)	Systematic review and meta-analysis	ICI-induced CV irAEs	[19,127,128]
ICI therapy + VEGF inhibitors (axitinib)	Phase 3 clinical trial and meta-analysis	ICI-induced myocarditis	[130,131]

CAR-T: chimeric antigen receptor T; LV: left ventricle; BTE: bispecific T-cell engager; IL-6: interleukin-6; SVT: supraventricular tachycardia; CRS: cytokine release syndrome.

## 7. Follow-Up and Management

Cardiovascular toxicity risk is a dynamic parameter that evolves throughout the course of cancer and is influenced by both modifiable and non-modifiable factors. Accordingly, a thorough baseline assessment of cardiovascular toxicity risk is essential for guiding cancer treatment selection, formulating personalized preventive strategies, and ensuring effective therapeutic monitoring. A cardiovascular toxicity risk assessment should be performed using a methodology where all risk factors are incorporated. Although prospective validation is warranted, the European Society of Cardiology (ESC) guidelines endorse the use of the recently developed baseline risk stratification score by the Heart Failure Association (HFA) and the International Cardio-Oncology Society (IC-OS) [132,133,134]. All patients should have baseline ECG, troponin, and natriuretic peptide (BNP or NT-proBNP) measurements before the initiation of ICI, CAR-T, and tumor-infiltrating lymphocyte (TIL) therapy [49,135,136,137]. High-CTR cardiovascular toxicity-risk patients should have, in addition, an echocardiography evaluation, including LVEF, preferably by Simpson’s biplane method and GLS. For both primary and secondary prevention of cancer therapy-related cardiovascular toxicity, it is recommended to optimize modifiable cardiovascular risk factors through lifestyle interventions, including smoking cessation, limiting alcohol intake, and maintaining regular physical activity. Additionally, comprehensive management of pre-existing or emerging cardiovascular disease in accordance with relevant ESC guidelines is advised before, during, and after oncologic treatment [138]. Once therapy is initiated, patients should undergo regular follow-up with electrocardiography (ECG) and monitoring of cardiac biomarkers, including cardiac troponins (cTn) and natriuretic peptides [139,140,141]. The European 2022 guidelines recommend regular ECG and troponin monitoring during ICI therapy (particularly early cycles) and structured risk-based follow-up ASCO, but the AHA/ACC statements are less prescriptive about universal troponin surveillance; many U.S. summaries focus on surveillance when symptomatic/abnormal or per local protocols. Particular consideration should be given to the issue of polypharmacy, which is commonly observed in oncology patients. Efforts should be made to limit the use of non-essential medications, especially those with the potential to interfere with anticancer therapies, while ensuring vigilant monitoring for cardiovascular adverse effects and clinically significant drug–drug interactions [142]. Patients receiving immune checkpoint inhibitor (ICI) therapy are at an increased risk of developing myocarditis and, therefore, should be closely monitored for related clinical symptoms and signs. Weekly assessment of cardiac troponin levels is recommended for at least the first six weeks of treatment to facilitate early detection and appropriate management [63]. Immune-related cardiovascular irAEs most commonly occur within the first four cycles of immunotherapy; however, approximately 25% of cases may manifest after the fourth treatment cycle [28]. Therefore, follow-up with ECG and cTn measurements should be considered prior to the administration of the second, third, and fourth doses of immune checkpoint inhibitors (ICIs). If results remain within normal limits, monitoring frequency may be reduced to every three doses for the remainder of the therapy [143]. However, in high-risk patients requiring long-term immune checkpoint inhibitor (ICI) treatment, regular evaluations—including physical examination, blood pressure measurement, natriuretic peptides, lipid profile, HbA1c, and ECG—are recommended every 6 to 12 months [40,51,144,145,146]. On the other hand, patients undergoing CAR-T cell therapy should undergo cardiovascular evaluation, including ECG, cardiac biomarkers, and transthoracic echocardiography (TTE), seven days following CAR-T cell infusion and subsequently followed up by evaluation at three months post-treatment. Additional evaluation with cardiac magnetic resonance imaging (CMR) should be performed if abnormal findings are observed in an asymptomatic patient [147]. In patients on CAR-T therapy, CRS should be highly suspected when symptoms such as fever, tachypnoea tachycardia, hypotension, hypoxia, and/or other end-organ dysfunction develop hours to days after receiving CAR-T therapy. It is imperative to differentiate CRS from other conditions such as infections, pulmonary embolism, or drug-related adverse reactions [148,149].

**Figure 1:**
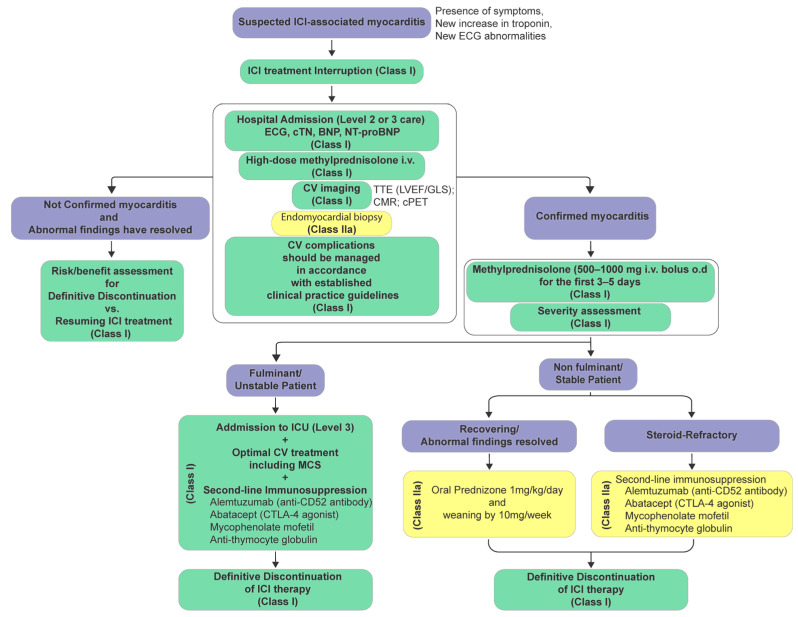
Proposed flowchart for the diagnosis, assessment, and management of immune checkpoint inhibitor-related myocarditis according to the European guidelines [138]. Class of recommendation indicates how strongly a particular treatment, procedure, or intervention is recommended based on the available evidence. Briefly, Class I (strong recommendation): the proposed action is recommended or is indicated; Class II (moderate recommendation) is subdivided into IIa and IIb; Class IIa: Evidence/opinion leans in favor; Class IIb: Evidence/opinion is less well established; Class III (no benefit or harm), action is not recommended [138]. ICI: immune checkpoint inhibitor; CV: cardiovascular; cTn: cardiac troponin; BNP: B-type natriuretic peptide; NT-proBNP: N-terminal pro-brain-natriuretic-peptide; TTE: transthoracic echocardiography; LVEF: left ventricular ejection fraction; GLS: global longitudinal strain; CMR: cardiac magnetic resonance imaging; cPET: cardiac positron emission tomography; ICU: intensive care unit; MCS: mechanical circulatory support; CTLA: cytotoxic T lymphocyte-associated antigen-4.

Cardiotoxicity due to immunotherapy may be life-threatening; therefore, a high suspicion for identification and management of cardiac irAEs as early as possible is very important. In patients currently undergoing or recently treated with ICIs, the emergence of new cardiovascular symptoms or the incidental identification of arrhythmias or conduction disturbances necessitates immediate clinical assessment. In cases of acute pericarditis, patients typically present with chest pain (>85% to 90% of cases), mainly sharp and pleuritic, which augments in the supine position with cough and deep inspiration, while it subsides in the upright position and by leaning forward. This pleuritic pain usually radiates to the trapezius ridge of the left shoulder or arm [150]. Other typical manifestations include isolated pericardial effusion and cardiac tamponade [37,151,152,153]. The clinical manifestations of ICI-related myocarditis range from asymptomatic cardiac biomarker elevation or mild, non-specific symptoms to chest pain, dyspnea due to acute heart failure and pulmonary edema, cardiogenic shock, and severe arrhythmias or sudden death [31,41,154,155]. Both European and American guidelines strongly recommend immediate assessment with ECG, cardiac troponin, BNP or NT-proBNP, transthoracic echocardiography, and CMR (Figure 1). A TTE assessment includes both LVEF and GLS analysis [117,156]. Emerging data show that global longitudinal strain obtained together with 2-D echocardiography may be helpful both for diagnosis and for prognosis [157]. According to the ESC guidelines 2022, clinical diagnosis of “definitive myocarditis” may be established either by the presence of positive endomyocardial biopsy or new increase or significant change in troponin values from baseline associated with either:

a. CMR diagnostic for myocarditis (Lake-Louise Criteria) [158] or

b. two of any of the following criteria:

(i) clinical symptoms (fatigue, muscle weakness, myalgias, chest pain, diplopia, ptosis, shortness of breath, orthopnea, lower extremity edema, palpitations, lightheadedness/dizziness, syncope or cardiogenic shock);

(ii) ventricular arrhythmia and/or new conduction system disease;

(iii) a decline in cardiac systolic function (reduction in left ventricular ejection fraction [LVEF] from baseline by ≥5% to <55% in the presence of signs or symptoms of HF, or a reduction in LVEF by ≥10% to <55% without signs or symptoms of HF, or LVEF < 50% in cases of non-available baseline transthoracic echocardiogram) [159,160].

CMR is also effective in demonstrating myocardial inflammation and necrosis by T1 and T2 sequences and late gadolinium enhancement [17]. Although endomyocardial biopsy it is the gold standard for ICI-related myocarditis verification, due to safety and convenience, endomyocardial biopsy is reserved for patients who do not respond to initial treatment or in cases of doubtful diagnosis [161].

When a diagnosis is confirmed, risk stratification should be established. According to the Society for Immunotherapy of Cancer (SITC), four grades of cardiovascular irAEs have been recommended [162].

Grade 1 is defined as abnormal cardiac biomarkers or abnormal ECG findings in asymptomatic patients. These patients need close monitoring during therapy;

Grade 2 is defined as patients with mild symptoms associated with abnormal screening tests. These patients are managed by holding ICIs and treating–controlling coexisting cardiac disease, as well as related risk factors such as hypertension or hyperlipidemia;

Grade 3 is defined as at least mild active symptoms or moderately abnormal testing. These patients are managed by initiating immediate high-dose corticosteroids and evaluation of discontinuing ICIs;

Grade 4 is defined as moderate to severe decompensated cardiac impairment and life-threatening conditions. These patients are managed by intravenous high-dose corticosteroids and discontinuation of ICIs; and

**Figure 2:**
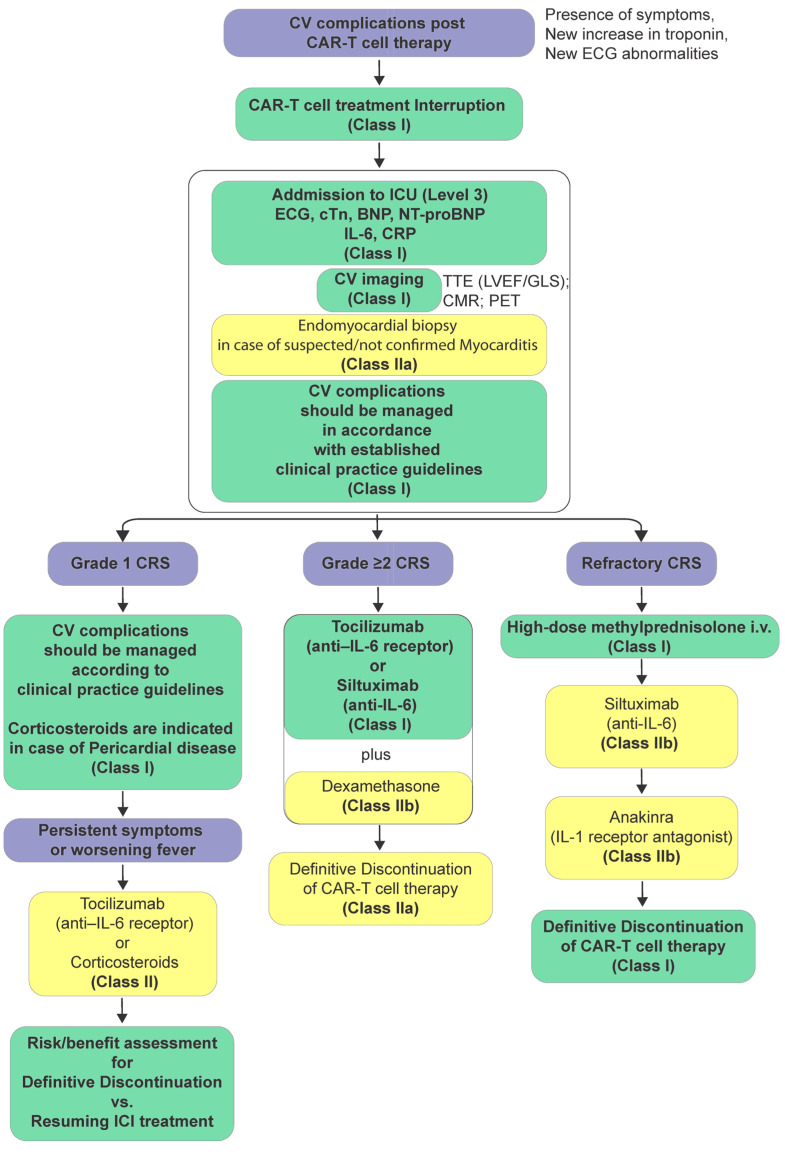
Proposed flowchart for the diagnosis, assessment, and management of CAR-T cell-related CV-AEs [138]. Class of recommendation indicates how strongly a particular treatment, procedure, or intervention is recommended based on the available evidence. Briefly, Class I: (strong recommendation) the proposed action is recommended or is indicated; Class II (moderate recommendation) is subdivided into IIa and IIb; Class IIa: Evidence/opinion leans in favor; Class IIb: Evidence/opinion is less well established; Class III (no benefit or harm), action is not recommended [138]. CAR-T cell: chimeric antigen receptor-T cell; CV: cardiovascular; cTn: cardiac troponin; BNP: B-type natriuretic peptide; NT-proBNP: N-terminal pro-brain-natriuretic-peptide; TTE: transthoracic echocardiography; LVEF: left ventricular ejection fraction; GLS: global longitudinal strain; CMR: cardiac magnetic resonance imaging; cPET: cardiac positron emission tomography; ICU: intensive care unit; CRS: cytokine release syndrome; IL-6: interleukin-6; IL-1: interleukin-1.

Grade 5 is defined as death due to cardiovascular toxicity [162,163]. In cases of immune checkpoint inhibitor (ICI)-associated myocarditis, both European [138] and American [164] guidelines recommend immediate interruption of ICI therapy. This is because of the concern for high mortality related to this cardiovascular adverse event. Other causes of troponin elevation should be ruled out, including ACS if appropriate, especially in patients with cardiovascular risk factors or established coronary artery disease presenting with chest pain and elevated troponin [156]. Prompt initiation of high-dose methylprednisolone is advised, particularly in hemodynamically unstable patients, with subsequent monitoring through imaging [43]. All cases should be classified based on myocarditis severity to guide the management strategy [140], while cardiovascular complications should be managed in accordance with the relevant ESC guidelines (Figure 1) [165,166,167]. Both American and European guidelines agree on initiation of high-dose corticosteroids and escalation if there is no response. Treatment of both non-fulminant and fulminant ICI-associated myocarditis should be initiated as early as possible—once the diagnosis is considered likely—with intravenous methylprednisolone at a dose of 500–1000 mg once daily for the first 3 to 5 days in order to reduce the risk of major adverse cardiovascular events (MACEs), including mortality [43,148]. In case of clinical improvement—defined by a ≥50% reduction in cardiac troponin levels from peak within 24 to 72 h, along with resolution of left ventricular systolic dysfunction, atrioventricular (AV) block, and arrhythmias, transition to oral prednisolone is recommended, initiated at 1 mg/kg (up to a maximum of 80 mg/day). Conversely, if the troponin level begins to increase again or if troponin levels fail to decline significantly (i.e., <50% reduction from peak) and/or AV block, ventricular arrhythmias, or LVD persist despite 3 days of intravenous methylprednisolone and adjunctive cardiac therapies, the diagnosis of steroid-resistant ICI-associated myocarditis should be considered, warranting escalation to second-line immunosuppressive therapy [117,168,169] or increasing steroid dosing and tapering over a longer period [170]. The safety of reinitiating immune checkpoint inhibitor (ICI) therapy following an episode of successfully treated ICI-related myocarditis remains uncertain. A case report describing subsequent initiation of pembrolizumab after an episode of ICI-associated myocarditis while receiving nivolumab led to worsening heart failure within two weeks of the first dose, necessitating hospitalization and permanent discontinuation of immunotherapy [171]. Given the potential for recurrent and severe cardiotoxicity, re-challenge with ICI therapy in patients with a history of ICI-associated myocarditis is not currently recommended. ASCO/SITC recommend holding immune checkpoint inhibitor therapy starting with Grade 1 cardiotoxicity (asymptomatic biomarker elevation or imaging abnormality) and permanently discontinuing for grade 2 or higher [17], while the ESC advises a more nuanced and conservative approach—interrupt at suspicion, and after confirmed myocarditis, typically avoid re-challenge, especially if moderate–severe; any consideration is highly individualized in a multidisciplinary team context and remains evidence-poor [172]. Treatment of ICI-associated pericarditis consists of the use of non-steroidal anti-inflammatory drugs (NSAIDs) and colchicine, and corticosteroids if needed. Pericardiocentesis for the large effusions is indicated, and for the steroid-resistant cases, successful results were reported with other treatments like mycophenolate and TNF-alpha inhibitors [173]. Based on the available evidence, ICIs could be continued for Grade 1 pericarditis, while temporary suspension of ICIs is recommended for Grade 2 to Grade 4 [174]. In case of cardiovascular (CV) complications in patients receiving CAR-T cell therapy, treatment interruption is also recommended, and patients should be admitted to an intensive care unit (ICU) for continuous electrocardiographic monitoring, given the high incidence of arrhythmias associated with CAR-T therapy, including QTc prolongation and ventricular arrhythmias [94]. Elevated interleukin-6 (IL-6) levels support the diagnosis of CRS (Table 4). Management should include standard treatment of CV complications in accordance with clinical guidelines. Mild CRS is frequently manageable with supportive measures alone [147]. However, in moderate to severe cases in patients who develop Grade ≥ 2 CRS (Table 4), targeted inhibition of the interleukin-6 (IL-6) signaling pathway is recommended. This can be achieved using tocilizumab, a monoclonal antibody directed against the IL-6 receptor, or siltuximab, an interleukin-6 inhibitor, which binds directly to soluble IL-6 (Figure 2), thereby mitigating its pro-inflammatory effects [147]. Additionally, dexamethasone should be added in case of myocarditis or pericarditis or when there is no improvement after tocilizumab [147]. If there is no clinical improvement within 12–24 h following tocilizumab administration, or in the event of clinical deterioration, initiation of corticosteroid therapy—such as dexamethasone or methylprednisolone—is warranted. Moreover, in cases of inadequate response to repeated doses of tocilizumab and corticosteroids, or in the presence of severe or refractory CRS, particularly with cardiovascular or neurological involvement, escalation to second-line agents—such as siltuximab or the interleukin-1 receptor antagonist Anakinra [91,138,175]. To date, administration of IL-6 receptor antagonists and/or corticosteroid treatment has not been associated with an increased incidence of cancer recurrence [176]. Therefore, in many centers, the standard approach involves the use of tocilizumab as the first-line treatment for patients presenting with grade 2 or higher CRS, with corticosteroids introduced as a second-line therapy when tocilizumab proves insufficient [177,178].

## 8. Precision Cardio-Oncology

Recent studies in cardio-oncology have focused on developing personalized strategies to predict susceptibility to cardiotoxicity and mitigate cardiovascular toxicity in cancer patients undergoing immunotherapy. Such strategies integrate artificial intelligence (AI) and machine learning [179], genetic and transcriptomic profiling [180,181], novel biomarkers, and development of clinical risk scoring to improve patient care. AI has become an essential component of modern medicine, providing innovative and powerful solutions for early detection of cardiotoxicity, risk stratification, and the optimization of therapeutic interventions [179,182,183]. Dr. Heilbroner and colleagues, using the CancerLinQ database curated by the American Society of Clinical Oncology and applying the XGBoost machine learning model, aimed to predict cardiac events in patients treated with PD-1/PD-L1 inhibitors [184]. This study included 4960 patients who were treated with anti-PD-1/PD-L1 therapy, of whom 418 had a cardiac event. A total of 356 potential risk factors were encompassed in the model, including patient history, laboratory tests, and treatment specifics, achieving an AUC of 0.65 (95% CI: 0.58–0.75). Key predictors identified were age, corticosteroid use, lymphocyte and neutrophil percentages, and weight extremes. Another study examined an AI-enhanced electrocardiograph (AI-ECG) to stratify risk for chemotherapy or immunotherapy-related cardiotoxicity. In patients receiving anthracyclines or trastuzumab, a positive AI-ECG screen correlated with a 2-fold increase in cardiotoxic events and a 4.8-fold increase in left ventricular ejection fraction dropping below 40% [185]. Another investigation applied deep learning models, such as convolutional neural networks (CNNs), to classify patients who developed irAEs based on their clinical narratives and determine whether there is evidence that a patient developed an irAE [186]. Another study presented a predictive model that incorporated both breast cancer treatment-related risk factors, such as hypertension and diabetes, to estimate the likelihood of major adverse cardiac events over a period of up to seven years [187]. A different risk prediction model used data from 30,286 low-dose CT scans from patients with lung cancer; it effectively identified patients at high risk for increased cardiovascular disease mortality and demonstrated superior performance compared to existing models [188].

In addition, human genotyping provides additional opportunities to further identify cancer patients at risk for cardiovascular toxicities [189]. Genomic studies aimed at identifying single-nucleotide polymorphisms (SNPs) associated with cardiotoxicity [190,191], along with transcriptomic profiling using bulk or single-cell RNA sequencing (scRNA-seq) [192], hold promise for uncovering novel biological mechanisms and improving the prediction of cardiac adverse events in immunotherapy. Although investigations into immunotherapy-induced cardiotoxicity remain limited, several genetic variants have already been implicated in susceptibility to chemotherapy-induced cardiotoxicity, highlighting the potential of precision medicine approaches in this field [193,194,195,196]. Polymorphisms in the CTLA-4 gene, such as rs4553808, rs11571317, and rs231775, have been associated with differential responses to ipilimumab therapy and the occurrence of immune-related endocrine adverse effects. Although these variants have not been directly associated with cardiotoxicity, they may contribute to the overall risk of irAEs and provide valuable insights into genetic factors that may influence susceptibility to immunotherapy-associated complications [197]. In the field of transcriptomics, Gergely et al. employed RNA sequencing to assess the effects of anti-PD-1 therapy on cardiac tissue and identified interleukin-17A (IL-17A) as a critical mediator of cardiac dysfunction, suggesting that IL-17A-driven pathways may underlie immune checkpoint inhibitor (ICI)-induced cardiotoxicity [198]. Moreover, genetic variants in the IL7 gene have been implicated in heightened susceptibility to ICI-associated cardiac injury, further supporting the role of host genomic factors in modulating risk [199]. In another study, transcriptomic profiling of myocardial tissue from patients with ICI-related myocarditis revealed upregulation of immune-related genes, including IL6R, HLA-C, HLA-E, STAT2, and IKBKB, and downregulation of genes such as DDX43 and HMGB1 [200]. Pathway enrichment analyses highlighted the involvement of cytokine-mediated signaling and Th17 cell differentiation, underscoring the contribution of dysregulated immune gene expression to ICI-induced myocarditis [200]. Given these findings, both American and European guidelines emphasize the importance of comprehensive cardiovascular evaluation prior to initiating immunotherapy, particularly in patients with pre-existing cardiovascular disease [117,156,201]. Baseline cardiovascular imaging with TTE is recommended, with CMR imaging as an alternative in cases of suboptimal echocardiographic quality. These modalities are critical for early detection, diagnostic confirmation, and longitudinal monitoring of cardiotoxicity. Clinically significant findings include a reduction in LVEF of more than 10 percentage points to a value below 50%, or a drop of more than 20 percentage points irrespective of baseline, or an absolute GLS decrease exceeding 5%, or a relative GLS reduction ≥ 12%, all considered abnormal and indicative of the need for initiation of cardioprotective therapy [117,202]. Moreover, elevations in native T1 on CMR have shown strong predictive value for future MACEs [154], while native T1, extracellular volume (ECV), and late gadolinium enhancement (LGE) have also demonstrated prognostic utility for survival outcomes in this population [155]. Emerging data from ongoing clinical trials are expected to further refine risk stratification and management strategies [203].

## 9. Discussion

Cardio-oncology is an increasingly recognized interdisciplinary field that aims to enable patients with cancer to undergo effective cancer treatments safely, with careful attention to cardiovascular health. It involves the evaluation and management of cardiovascular risk and disease before, during, and after cancer therapy, addressing both short- and long-term cardiovascular considerations. With immunotherapy transforming the landscape of cancer treatment by significantly extending survival across a wide range of malignancies and diverse patient populations, the need for accurate cardiovascular risk assessment, early detection of cardiotoxic effects, and prompt therapeutic intervention becomes increasingly critical. Both American and European guidelines underscore the necessity of comprehensive cardiovascular evaluation prior to initiating immunotherapy, especially in individuals with pre-existing cardiovascular disease. A comprehensive history intake and efficient physical exam are crucial and may help to facilitate an early and accurate diagnosis. Our review findings are aligned with both the ESC and AHA recommendations, particularly regarding baseline cardiovascular assessment, multimodality imaging, and longitudinal surveillance in high-risk groups. Notably, the ESC guidelines are more proactive in recommending systematic surveillance with ECG and troponin testing during the early cycles of immunotherapy, whereas American guidelines are less prescriptive and focus surveillance primarily on symptomatic patients or those with abnormal findings. In contrast, both sets of guidelines converge on the urgent initiation of high-dose corticosteroids in suspected myocarditis and emphasize multidisciplinary team (MDT) involvement in complex cases.

Despite growing awareness, several important knowledge gaps remain that warrant further investigation. These include: (a) the identification of more effective and widely accessible methods for screening and diagnosing cardiovascular toxicities; (b) improved risk stratification and implementation of targeted surveillance and preventive strategies; and (c) the development of optimized management pathways that balance effective oncologic treatment with cardiovascular safety in patients with established cardiotoxicity. Current conventional biomarkers alone are insufficient for comprehensive cardiovascular risk prediction. Emerging research suggests that omics-based technologies—including genomics, transcriptomics, proteomics, and metabolomics—may yield novel, sensitive, personalized biomarkers and help define cardiovascular risk thresholds for existing markers in clinical practice. In the future, integrating data from multiple omics platforms may refine cardiovascular risk stratification, improve early detection, and guide personalized interventions. In addition, multimodality cardiovascular imaging will remain a critical component, complementing biomarkers and clinical evaluation in the assessment of cardiac toxicities. GLS has emerged as a sensitive and reliable echocardiographic parameter for the early detection of subclinical myocardial dysfunction, often preceding measurable changes in LVEF. GLS plays an essential role in guiding timely cardioprotective strategies and is endorsed by current guidelines for risk assessment and monitoring. Despite some technical limitations, GLS-based surveillance has demonstrated clinical utility and is increasingly being adopted into routine cardio-oncology practice. However, real-world application of advanced diagnostics—including GLS, multimodality imaging, and biomarker assays—is often constrained by cost, availability, and the need for specialized expertise. CMR may not be widely accessible, and inter-laboratory variability or patient-specific factors can influence biomarker interpretation, emphasizing the importance of integrating these tools with clinical assessment and other imaging modalities. Ongoing development of advanced imaging modalities and techniques for detecting treatment-induced cardiac injury will further enhance patient care.

In parallel, recent advances in artificial intelligence (AI) and machine learning, as well as genotyping and transcriptomic profiling, provide additional opportunities to further identify cancer patients at risk for cardiovascular toxicities and improve diagnostic accuracy, therapeutic planning, and, therefore, clinical outcomes in cardiovascular care. These technologies hold the potential to generate individualized cardiotoxicity risk models by incorporating patient-specific data, clinical data, imaging findings, and biomarker trajectories collected during follow-up. Although these applications are still in the early stages of development, AI-driven tools are expected to become progressively integrated into routine clinical workflows to support personalized care. Based on the evidence synthesized in this review, we proposed algorithms that are in accordance with the ESC guidelines. Their purpose is to operationalize existing recommendations into practical, step-by-step approaches for clinicians, ensuring that guideline principles can be applied consistently across diverse clinical contexts. These algorithms also provide structured guidance in emerging areas—such as the monitoring of patients treated with anticancer vaccines or the integration of omics-based biomarkers and AI tools—where standardized protocols are not yet available. Thus, our proposed algorithms complement rather than replace current ESC and AHA recommendations, aiming to fill gaps in clinical application.

This integrative, multiparametric approach—combining clinical evaluation, imaging, biomarkers, omics data, and AI—holds substantial promise for reducing cardiovascular morbidity and mortality in cancer patients and survivors. Ultimately, such innovations are poised to drive significant advancements in the evolving discipline of cardio-oncology.

## 10. Conclusions

Cancer immunotherapy has transformed oncology by offering unprecedented survival benefits across a wide spectrum of malignancies. However, its potential for immune-mediated cardiovascular adverse events—including myocarditis, heart failure, arrhythmias, pericarditis, and accelerated atherosclerosis—poses significant challenges to patient safety. Our review highlights the critical importance of a proactive, personalized approach to cardio-oncology, integrating baseline cardiovascular risk assessment, multimodality imaging, and longitudinal surveillance.

Emerging strategies—including the use of global longitudinal strain (GLS), omics-based biomarkers, and artificial intelligence-driven risk modeling—offer promising avenues for early detection, precise risk stratification, and individualized management of cardiotoxicity. Multidisciplinary collaboration between oncologists, cardiologists, and allied healthcare professionals remains essential to optimize outcomes and ensure safe delivery of life-saving immunotherapies.

Despite these advances, important knowledge gaps remain. Future research should focus on refining diagnostic algorithms, validating novel biomarkers, and developing standardized, cost-effective surveillance protocols that balance oncologic efficacy with cardiovascular safety. Ultimately, integrating clinical evaluation, advanced imaging, biomarker profiling, and AI-driven tools represents the frontier of precision cardio-oncology, with the potential to significantly reduce cardiovascular morbidity and mortality in cancer patients while enabling the full therapeutic potential of immunotherapy. However, these strategies need more data and clinical trials to be validated and standardized in order to be used in clinical practice.

## Figures and Tables

**Table 2:** Chimeric antigen receptor T-cell therapy-associated cardiovascular adverse events.

Cancer Therapy	Cardiovascular Adverse Events	Key Findings—Comments	Study
CD19-directed CAR-T	Arrhythmias: 77.6%; heart failure: 14.3%; myocardial infarction: 0.5%	30.1% mortality among those with CVEs	[94]
CD19-directed CAR-T	Cardiogenic shock–death: 4%; new-onset arrhythmia: 4%	From 137 patients, 6 developed shock leading to cardiac mortality	[87,95]
CD19-directed CAR-T	Prolonged QTc		[95]
CD19-directed CAR-T	Tachyarrhythmias: 2.8%; cardiomyopathy: 2.6%; pericardial diseases: 0.4%	Atrial fibrillation most common; ventricular arrhythmias less common	[98]
	Supraventricular arrhythmias: 7.79%; ventricular arrhythmias: 0.66%; left Ventricular systolic dysfunction: 8.68%; heart failure: 3.87%; myocardial infarction: 0.62%	Cardiovascular death: 0.63%; all-cause mortality: 30.01%	[99]
	Heart failure: 11–15%; arrhythmias: 1.4–12%	IL-6 implicated; CRS severity linked to CVEs	[97]
CD19-Specific CAR-T	LV dysfunction: 2–11%; myocarditis: 31% of CRS patients	Pediatric patients also at risk of cardiotoxicity	[87,100,101,105,106]
	Shock: 9–37%		[100,105,106,107,108,109,110]
	Sinus tachycardia; ST-segment changes: 18%		[105,106]
	CRS: 58–100%		[87,100,105,107,108,109,110]
TCR-engineered T cells	Cardiogenic shock–death	Off-target toxicity due to cross-reactivity with titin	[82]
BTE	Arrhythmias: 1.67–14%; LV dysfunction second most common	Supraventricular arrhythmias are predominant	[102,103,104]

CAR-T: chimeric antigen receptor T; LV: left ventricle; BTE: bispecific T-cell engagers; IL-6: interleukin-6; SVT: supraventricular tachycardia; CRS: cytokine release syndrome.

**Table 4:** Grading for cytokine release syndrome according to the American Society for Transplantation and Cellular Therapy [91].

Parameter	Grade 1	Grade 2	Grade 3	Grade 4
**Fever** (≥38 °C) Not attributable to any other cause.	+	+	+	+
Plus				
**Hypotension**	None	Not requiring vasopressors	Requiring one vasopressor with or without vasopressin	Requiring multiple vasopressors (excluding vasopressin)
And/or				
**Hypoxia**	None	Nasal cannula (≤6 L/minute) or blow-by	High-flow nasal cannula (>6 L/minute), facemask, nonrebreather mask, or Venturi mask	Positive pressure (CPAP; BiPAP; intubation mechanical ventilation)

## Data Availability

Not applicable.

## Supplementary Materials

The following supporting information can be downloaded at: https://www.mdpi.com/article/10.3390/cancers17172838/s1, Table S1. Detailed studies. References [18,25,26,28,31,51,56,87,95,96,97,98,99,100,101,106,110,204,205,206,207,208,209,210] are cited in the Supplementary Materials.

## Abbreviations

The following abbreviations are used in this manuscript:

ICI	Immune Checkpoint Inhibitor
CAR-T	Chimeric Antigen Receptor T-Cell
CRS	Cytokine Release Syndrome
PD-1	Programmed Cell Death 1
PD-1L	Programmed Cell Death 1 Ligand
CTLA-4	Cytotoxic T-Lymphocyte Antigen 4
LAG3	Lymphocyte Activation Gene-3
BTE	Bispecific T-Cell Engagers
irAEs	Immune-Related Adverse Events
LVEF	Left Ventricular Ejection Fraction
GLS	Global Longitudinal Strain
IL-6	Interleukin-6
TNF-α	Tumor Necrosis Factor-A
IFN-γ	Interferon Gamma
IL-1	Interleukin-1
MI	Myocardial Infraction
HF	Heart Failure
ESC	European Society of Cardiology
HFA	Heart Failure Association
IC-OS	International Cardio-Oncology Society (Ic-Os)
ECG	Electrocardiography
BNP	B-Type Natriuretic Peptide
NT-proBNP	N-Terminal Pro-Brain-Natriuretic-Peptide
cTn	Cardiac Troponin
HbA1c	Glycated Hemoglobin
CMR	Cardiac Magnetic Resonance Imaging
LGE	Late Gadolinium Enhancement
TTE	Transthoracic Echocardiography
AV	Atrioventricular
ICU	Intensive Care Unit
AI	Artificial Intelligence
SNPs	Single-Nucleotide Polymorphisms
cPET	Cardiac Positron Emission Tomography
MACE	Major Adverse Cardiovascular Events
