# Supplementary material for: Immunotherapy-Associated Cardiotoxicity: Current Insights and Future Directions for Precision Cardio-Oncology
Source: Cancers (Basel). 2025 Aug 29;17(17):2838. doi: 10.3390/cancers17172838 (PMC12427380; doi:10.3390/cancers17172838)
Supplement: Supplementary file 1 [file cancers-17-02838-s001.zip › cancers-3769044-supplementary.pdf]

Supplementary Table S1. Detailed studies

Major studies of Immune checkpoint inhibitors-related cardiotoxicities

Study	Type of study	No. of patients	Treatment	Cardiovascular adverse events	Onset days/ Mean range	Outcomes
Drobni et al., [18]	Single-centre case-control	2842	ICI treatment	Myocardial Infarction (0.95%)<br>Ischeamic Stroke (0.91%)	N/A	N/A
Moslehi et al., [25]	Retrospective study, WHO VigiBase pharmacovigilance	101	Anti-PD-1 monotherapy, anti-PD-L1 + anti-CTLA-4	Myocarditis	27/ range 5 to 155	Death for Monotherapy treatment (36%)<br>Death for combination treatment (67%)
Salem et al., [26]	Observational-Retrospective study, WHO VigiBase pharmacovigilance	442	Nivolumab, Pembrolizumab, Atezolizumab, Avelumab, Durvalumab, Ipilimumab, Tremelimumab	Myocarditis (28%)<br>Pericardial disease (21%)<br>Vasculitis (19%)<br>Supraventricular arrhythmias (50%)	30/ range 1 to 40<br>30/ range 0 to 330<br>55/ range 21 to 98<br>14/ range 1 to 925	Death (50%)/Cardiogenic shock (15.6%)<br>Death (21%)/Cardiogenic shock (3.2%)<br>Death (6.1%)/Cardiogenic shock (0%)<br>Death (23.9%)/Cardiogenic Shock (2%)
Mahmood et al., [28]	Retrospective and prospective study, Multicentre registry of sporadic cases	140	**Monotherapy** (Pembrolizumab, Nivolumab, Ipilimumab, Tremelimumab, Atezolizumab) **Combination** (Ipilimumab+ Nivolumab, Ipilimumab+ Pembrolizumab, Tremelimumab+ avelumab, Tremelimumab+ durvalumab)	Myocarditis for Combination (8%)<br>Myocarditis for Monotherapy (16%)	34/ range 21 to 75	Death (17%)<br>Cardiogenic shock (8.6%)<br>Cardiac arrest (2.9%)<br>Complete heart block (8.6%)<br>MACE (46%)

Escudier et al., [31]	Retrospective study,<br>Case series	30	Ipilimumab;<br>Nivolumab;<br>Pembrolizumab;<br>Atezolizumab,<br>Ipilimumab+<br>nivolumab	Myocarditis (79%)<br>Atrial fibrillation (30%)<br>Conduction disorder (17%)<br>Ventricular arrhythmia (27%)<br>Heart Failure (83%)<br>Takotsubo cardiomyopathy (14%)	65/ range 2 to 54	Death (26.7%)<br>Cardiac arrest (6.7%)
D'Souza et al., [51]	Retrospective cohort<br>study in four Danish<br>registries	1100	Ipilimumab,<br>Nivolumab,<br>Pembrolizumab	Myocarditis (≈1,4%)<br>Arrhythmia (≈4%)<br>Heart Failure (≈1.6%)	75/ range 34 to 149<br>174/ range 2 to 455<br>N/A	N/A<br>N/A<br>Death (3.6%)
Oren et al., [56]	Mayo Clinic database<br>registry	3326	Atezolizumab,<br>Avelumab,<br>Ipilimumab,<br>Nivolumab,<br>Pembrolizumab	Myocarditis (0.36%)<br>Pericardial disease (1.74%)<br>Vasculitis (0.27%)<br>Myocardial Infarction (7%)<br>Stroke (7%)	138/ range 18 to 138<br>195/ range 3 to 1143<br>243/ range 6 to 1026<br>N/A<br>N/A	Death (42%)<br>Death (26%)<br>Death (0%)<br>N/A<br>N/A

Major studies of chimeric antigen receptor T-cell and bispecific T-cell engager-related cardiotoxicities

Study	Type of study	No. of patients	Treatment	Cardiovascular adverse events	Onset days/Mean range	Outcomes
Alvi et al., [87]	Retrospective cohort<br>study	137	CD19-directed<br>CAR-T	Atrial fibrillation (2.2%)<br>Arrhythmia (5.1%)<br>Heart Failure (5,8%)	21/ range 11 to 38	Death (4.3%)<br>Cardiac arrest (2.2%)<br>CRS grade 1-4 (59%)<br>CRS grade 3-4 (4%)
Lee et al., [95]	Open label<br>phase 1 trial	21	CD19 CAR T-cell	QTc (5.0%)<br>Heart Failure (5.0%)<br>Hypertenssion (5.0%)	range 1 to 7	Cardiac arrest (5.0%)<br>CRS grade 1-4 (76%)<br>CRS grade 3-4 (29%)
Lefebvre et al., [96]	Retrospective study	145	CD19-directed<br>CAR-T	Atrial fibrillation (7.6%)<br>Arrhythmia (9%)<br>ACS (1.4%)<br>Heart Failure (15%)	11/ range 6 to 151	Death (1.4%)<br>Cardiogenic shock (50%)<br>Cardiac arrest (0.0%)<br>CRS grade 1-4 (72%)<br>CRS grade 3-4 (N/A%)
Ganatra et al., [97]	Retrospective cohort<br>study	187	CD19-directed<br>CAR-T	Arrhythmia (7%)<br>Heart Failure (10.3%)	N/A	Cardiogenic shock (7%)<br>Cardiac arrest (0.0%)<br>CRS grade 1-4 (83%)<br>CRS grade 3-4 (5.3%)

Maude et al., [100]	Single-center phase 1–2a study, NCT02435849	75	Tisagenlecleucel (CD19-directed CAR-T)	Heart Failure (2.7%)	N/A	Cardiogenic shock (25%)<br>Death (4.0%)<br>CRS grade 1-4 (77%)<br>CRS grade 3-4 (46%)
Shalabi et al., [101]	Phase I trial (NCT01593696)	52	CD19-directed CAR T-cell	Heart Failure (11.5%)	5/ range 1–12	Cardiac arrest (1.9%)<br>Cardiogenic shock (17.3%)<br>CRS grade 1-4 (17%)<br>CRS grade 3-4 (71%)
Goldman et al., [98]	Retrospective, pharmacovigilance study	2657	CAR-T (Axicabtagene-ciloleucel and tisagenlecleucel)	Hypotension (10.8%)<br>Cardiomyopathy (2.6%)<br>Pericardial Diseases, 0.4%<br>Tachyarrhythmias (2.8%)	N/A	Cardiogenic shock (1.8%)<br>CRS grade 1-4 (54.84%)
Koeckerling et al., [99]	Meta-analysis Retrospective	1528	CD19-directed CAR T-cell	Ventricular Arrhythmia (0.66%)<br>Supraventricular Arrhythmia (7.79%)<br>Left Ventricular Dysfunction (8.68%)<br>Heart Failure (3.87%)<br>Myocardial Infarction (0.62%)	N/A	cardiovascular death (0.63%)
Burstein et al., [106]	Retrospective study, Case series	93	CD19-directed CAR-T	Heart Failure (10.8%)	4.6/ range 1 to 9	Cardiac arrest (1.1%)<br>Cardiogenic shock (15.6%)<br>CRS grade 1-4 (N/A%)<br>CRS grade 3-4 (25.8%)
Schuster et al., [110]	International, multicenter phase 2, pivotal study, NCT02445248	93	Tisagenlecleucel (D19-directed CAR-T)	N/A	7/ range 2 to 30	Death (0.0%)<br>Cardiogenic shock (26%)<br>CRS grade 1-4 (58%)<br>CRS grade 3-4 (21.5%)
Qi et al., [204]	Single-Centre Retrospective Study	126	CD19-; CD20-; BCMA-directed CAR-T	Arrhythmia (5.6%)<br>ACS (7.1%)<br>Heart Failure (11.9%)	5/ range 3 to 9	Death (1.6%)<br>Cardiac arrest (0.0%)<br>CRS grade 1-4 (81.7%)<br>CRS grade 3-4 (17.5%)
Brammer et al., [205]	Retrospective Study	90	D19-directed CAR-T	Myocarditis (2.2%)<br>Arrhythmia (12.2%)<br>Heart Failure (1.1%)	2.5 / range 0 to 9	CRS grade 1-4 (88.9%)<br>CRS grade 3-4 (16.3%)

Kantarjian et al., [206]	Multi-institutional randomized phase 3 trial	271	Blinatumomab, CD3/CD19 BiTE	Hypertenssion (6.4%)<br>Atrial fibrillation (0.4%)<br>Arrhythmia (0.8%)<br>ACS (0.4%)<br>Heart Failure (0.4%)	N/A	Cardiac arrest (0.4%)<br>CRS grade 1-4 (14.2%)<br>CRS grade 3-4 (4.9%)
Locke et al., [207]	Single-arm, multicentre, registrational trial	101	Axicabtagene ciloleucel (CD19)	Hypertenssion (16%)	N/A	Death (1.0%)<br>Cardiac arrest (1.0%)<br>Cardiogenic shock (17%)<br>CRS grade 1-4 (93%)<br>CRS grade 3-4 (11%)
Wang et al., [208]	Multicenter, phase 2 trial	68	Brexucabtagene autoleucel (CD19 CAR T-cell)	N/A	4/ range 1 to 9	Cardiogenic shock (22%)<br>CRS grade 1-4 (91%)<br>CRS grade 3-4 (15%)
Munshi et al., [209]	Single-group, phase 2 study	128	Idecabtagene vicleucel	N/A	range 1 to 12	Cardiogenic shock (1.0%)<br>CRS grade 1-4 (84%)<br>CRS grade 3-4 (5.0%)
Abramson et al., [210]	Multicenter seamless design study	269	Lisocabtagene maraleucel (CD19 CAR T-cell)	Hypertenssion (14%)	range 1 to 14	Death (0.3%)<br>Cardiogenic shock (3.0%)<br>CRS grade 1-4 (42%)<br>CRS grade 3-4 (2.0%)

CAR-T: Chimeric antigen receptor T; **CRS**: Cytokine release syndrome.
